# Risk score model for predicting mortality among patients with lung cancer

**DOI:** 10.3389/fmed.2024.1400049

**Published:** 2024-10-14

**Authors:** Youn Huh, Hae-Rim Kim, Hwa Jung Kim, Ki Young Son

**Affiliations:** ^1^Department of Family Medicine, Uijeongbu Eulji Medical Center, Eulji University, Gyeonggi-do, Republic of Korea; ^2^College of Natural Science, School of Statistics, University of Seoul, Seoul, Republic of Korea; ^3^Department of Preventive Medicine and Clinical Epidemiology and Biostatics, Asan Medical Center, University of Ulsan College of Medicine, Seoul, Republic of Korea; ^4^Department of Family Medicine, Asan Medical Center, University of Ulsan College of Medicine, Seoul, Republic of Korea

**Keywords:** lung cancer, mortality, risk score, development cohort, validation cohort

## Abstract

**Background:**

To develop an accurate mortality risk predictive model among patients with lung cancer.

**Methods:**

The development cohort included 96,255 patients with lung cancer aged ≥19 years, who underwent a Korean National Health Insurance Service health check-up from 2005 to 2015. The validation cohort consisted of 18,432 patients (≥19 years) with lung cancer from another region. The outcome was all-cause mortality between January 1, 2005, and December 31, 2020.

**Results:**

Approximately 60.5% of the development cohort died within a median follow-up period of 2.32 (0.72–5.00) years. Risk score was highest in participants aged ≥65 years, followed by those who underwent treatment, had a history of emergency room visits, and were current smokers. Participants treated by surgery had the lowest risk score, followed by combined surgery and chemotherapy, combined surgery and radiation therapy, women, and regular exercisers. The C statistic in the development and validation cohorts was 0.78 (95% confidence interval, 0.77–0.78) and 0.81 (95% confidence interval, 0.78–0.84), respectively.

**Conclusion:**

Advanced age, lung cancer stage, and treatment type were strong risk factors of mortality in lung cancer patients, while being a woman and exercise were preventive factors. These will aid in the prediction of mortality and management of lung cancer patients.

## Introduction

Lung cancer resulted in 2.2 million new cases and was the second most common cancer in 2020 worldwide (1). In the United States, approximately 118,000 new cases of lung cancer were estimated in 2022, and this malignancy was the second most common after prostate cancer in men and breast cancer in women (2). In Korea, the age-standardized incidence rate of lung cancer was 27.6 per 100,000, and lung cancer was the fourth most common type of cancer (3). Despite improvements in lung cancer treatments, the survival rate of lung cancer is lower than that of any other cancer (4). For example, the 5-year survival rate in Korea from 2014 to 2018 was 32% for lung cancer, despite being 66% for all cancers except thyroid (3). This figure was higher than that in the United States or United Kingdom, which was likely related to health insurance system in Korea (5). Moreover, a previous study found that risk factors of mortality among adults with lung cancer included advanced age, male sex, treatment with radiotherapy, organ failure, infection, and admission to the intensive care unit (6).

Methods for assessing risk can be useful in identifying and selecting which patients will attend additional healthcare facilities, be at high risk of mortality, and experience decreased body function (7–9). Risk stratification models not only help quickly detect and manage patients with a poor prognosis, such as hospitalization or mortality, but also prevent low-risk patients from becoming high-risk and improve the health status in moderate-risk patients (10).

Because lung cancer is such a prevalent disease worldwide, the management of this malignancy is vital. However, studies on the risk factors and risk score for lung cancer-related mortality are insufficient. Therefore, we aimed to create a mortality risk score model using a combination of mortality risk factors based on nationwide Korean cohort data. In addition, the validity of our model was tested using data from a cohort of patients with lung cancer from another region in Korea.

## Methods

### Study participants

We used data from the Korean National Health Insurance System (KNHIS), which represents Koreans. The KNHIS was instituted in 2000 as the only national health insurance system in South Korea and covers more than 97% of the Korean population. The KNHIS database was created for use by public health researchers and policy makers. Therefore, it retains extensive medical data, including demographic characteristics, health check-up data, disease diagnosis codes, treatments, and procedures based on medical claims according to the International Classification of Diseases 10th Revision (ICD-10) codes for the South Korean population. The KNHIS data have been utilized by qualified researchers submitting a study plan approved by official review committees since 2015.

Using this database, we initially identified 228,258 individuals diagnosed with lung cancer (ICD-10 codes C33 and C34) who underwent a national health check-up offered by the KNHIS from January 1, 2005, to December 31, 2015. We selected patients from Seoul as the development cohort and those from Busan and Gyeongsangnam-do as the validation cohort. There were no inherent differences in standard of care or quality of care between the development and validation cohorts, as they were supervised by the Health Insurance Review and Assessment Service. However, because the number of medical institutions and medical staff were concentrated in the two cohorts, which are big cities, our study defined the largest city in Korea as the development cohort and the next largest local region as the validation cohort. Those who lived in other regions (*n* = 66,337), individuals aged <19 years (*n* = 100), and those with missing data for any of the study variables (*n* = 47,134) were excluded. Finally, 114,687 individuals (96,255 in the development cohort and 18,432 in the validation cohort) were eligible for the study.

This study adhered to the principles of the Declaration of Helsinki and was approved by the Institutional Review Board of Uijeongbu Eulji Medical Center (IRB No: UEMC 2021–08-022). The requirement for informed consent was waived by the Institutional Review Board of Uijeongbu Eulji Medical Center due to the use of anonymized and de-identified data.

### Main outcome of study

The outcome of our study was all-cause mortality between January 1, 2005, and December 31, 2020.

### Covariates

The KNHIS database includes accurate demographic characteristics and lifestyle data, which were evaluated using standardized, self-administered questionnaires. The lowest 20% of the income range of the participants was classified as low income, and the remaining incomes were classified as non-low income. Smoking status was classified into two groups: non-smoker and current smoker. Individuals who consumed any alcohol on a weekly basis were classified as alcohol drinkers, and those who did not were classified as non-drinkers. Regular physical activity was defined as follows: moderate-intensity exercise, such as light walking for at least 5 days per week or high-intensity exercise, such as tennis for at least 3 days per week. Health examinations were conducted by qualified medical staff and included anthropometric and laboratory measurements. Anthropometric parameters included height, body weight, and waist circumference (WC), which were evaluated using standard protocols and equipment. The height of the participants was measured to the nearest 0.1 cm using a stadiometer. Body weight was measured to the nearest 0.1 kg on a balance scale, with the participants wearing only undergarments. The body mass index (BMI) was calculated by dividing the body weight (kg) by the height squared (m^2^). According to the definition of obesity by the Korean Society for the Study of Obesity, BMI was divided into three groups as follows: < 18.5, 18.5–24.9, and ≥ 25 kg/m^2^. Blood pressure was checked while the participants sat and had rested for at least 5 min. After overnight fasting, the participants underwent laboratory tests, including serum glucose and total cholesterol.

Chronic diseases were identified according to health examination results and medical claims for disease diagnoses and medication prescriptions. Chronic diseases were defined by the Office of the Assistant Secretary for Health before the diagnosis of lung cancer (11). A total of 20 chronic diseases were identified.

The type of treatment for lung cancer was divided into eight groups: none, chemotherapy, surgery, radiation, surgery and chemotherapy, surgery and radiation, chemotherapy and radiation, and surgery combined with chemotherapy and radiation. Emergency room visits were identified according to whether the participants visited the emergency room within 1 year of death or the last follow-up period.

### Statistical analysis

We performed all analyses using the SAS software (version 9.4; SAS Institute, Cary, NC, United States). We identified mortality risk factors by multivariate Cox proportional hazards analysis and calculated points proportional to the regression coefficient values to approximate scores. Model 1 was not adjusted, while Model 2 was adjusted for covariables with a *p*-value <0.05 in Model 1. A risk score was calculated for each individual, and the scores were classified as low-, moderate-, and high-risk for mortality. The optimal cut off were selected by calculating maximized log likelihood. The cutoff values of the risk groups were 7 points and 11 points. Risk scores for the validation cohort were calculated using the same method as that of the development cohort. For validation, we created a receiver operating characteristic (ROC) curve for the development and validation cohorts.

Kaplan–Meier survival curves for patients in each of the three risk groups were generated to show the risk of mortality for both the development and validation cohorts. The predictive accuracy of the risk scoring system was evaluated using the C statistic and by estimating the difference between the mortality probability of the high- and low-risk groups within 1 and 5 years.

## Results

### Baseline characteristics

Table 1 shows the baseline characteristics of the 114,687 eligible participants with lung cancer. Among them, the proportion of men was 69.1% (*n* = 79,242). The median follow-up period was 2.32 (0.72–5.00) years. The mean age was 66.1 ± 10.9 years, and men tended to be older than women (*p* < 0.001). Men were more likely than women to be current smokers, alcohol drinkers, and perform regular exercise (*p* < 0.001). Additionally, low income was slightly more common among women than men. Moreover, women had a slightly higher mean BMI and total cholesterol than men. In contrast, the mean systolic and diastolic blood pressure were higher in men than women (*p* < 0.001). The proportion of participants who did not undergo treatment for lung cancer was the highest among the treatment types for both men and women. In men, no treatment was followed by radiation therapy, surgery, and combined chemotherapy and radiation therapy. In women, no treatment was followed by surgery, combined chemotherapy and radiation therapy, and radiation therapy. The proportion of participants with ≥5 chronic diseases was >50% among both men and women, but higher in women (*p* < 0.001). Finally, men were more likely to visit the emergency room than women (*p* < 0.001).

**Table 1:** Baseline characteristics of study participants.

	Total	Sex		P-value
		Men	Women	
Sex (men)	79,242 (69.1%)			
Age (years)	66.1 ± 10.9	66.8 ± 10.3	64.7 ± 11.9	<0.001
Current smoker	33,161 (28.9%)	31,263 (39.5%)	1898 (5.4%)	<0.001
Alcohol drinker	22,942 (20.0%)	21,831 (27.6%)	1,111 (3.1%)	<0.001
Regular exercise	42,507 (37.1%)	30,230 (38.2%)	12,277 (34.6%)	<0.001
Income (low)	20,600 (18.0%)	13,798 (17.4%)	6,802 (19.2%)	<0.001
BMI (kg/m^2^)	23.3 ± 3.2	23.2 ± 3.1	23.7 ± 3.3	<0.001
Type of treatment				
None	43,762 (38.2%)	30,752 (38.8%)	13,010 (36.7%)	<0.001
Chemotherapy	9,062 (7.9%)	5,434 (6.9%)	3,628 (10.2%)	
Surgery	17,234 (15.0%)	11,100 (14.0%)	6,134 (17.3%)	
Radiation	18,628 (16.2%)	15,265 (19.3%)	3,363 (9.5%)	
Surgery + chemotherapy	2,456 (2.1%)	1,333 (1.7%)	1,123 (3.2%)	
Surgery + radiation	5,393 (4.7%)	4,285 (5.4%)	1,108 (3.1%)	
Chemotherapy + radiation	13,940 (12.2%)	8,587 (10.8%)	5,353 (15.1%)	
Surgery + chemotherapy + radiation	4,212 (3.7%)	2,486 (3.1%)	1,726 (4.9%)	
Number of chronic diseases				
0	2,664 (2.3%)	1,975 (2.5%)	689 (1.9%)	<0.001
1	7,039 (6.1%)	5,296 (6.7%)	1,743 (4.9%)	
2	11,073 (9.7%)	8,192 (10.3%)	2,881 (8.1%)	
3	13,865 (12.1%)	10,184 (12.9%)	3,681 (10.4%)	
4	15,378 (13.4%)	11,017 (13.9%)	4,361 (12.3%)	
≥5	64,668 (56.4%)	42,578 (53.7%)	22,090 (62.3%)	
Emergency room visit (Yes)	34,616 (30.2%)	24,842 (31.4%)	9,774 (27.6%)	<0.001

All values are presented as the number (percentage) or mean ± standard deviation (SD).

### Risk analysis and risk scoring system in the development cohort

Table 2 shows the mortality risk analysis and risk scores of the development cohort. Approximately 60.5% (*n* = 58,241) of the development cohort had died within a median follow-up period of 2.32 years. The mortality risk of women was lower than that of men [HR (95% CI) 0.72 (0.71–0.74)]. The risk in those aged ≥65 years and 45–64 years was 2.32 (2.19–2.45) and 1.30 (1.23–1.38) times greater than that in those aged 19–44 years, respectively. Current smokers had a 35% higher risk of mortality than non-smokers [1.35 (1.33–1.38)]. Additionally, alcohol drinkers had an 8% higher risk of mortality compared to non-drinkers [1.08 (1.06–1.10)]. Patients that participated in regular exercise [0.85 (0.83–0.86)] had a decreased risk of mortality compared to non-exercisers, while low income patients [1.07 (1.05–1.09)] had an increased risk of mortality compared to patients at other income levels. Obese participants had the lowest risk of mortality [0.90 (0.89–0.92)], while underweight participants had the highest risk of mortality according to BMI (1.16 [1.12–1.21]). Participants treated by chemotherapy [1.24 (1.21–1.28)], radiation therapy [1.25 (1.22–1.28)], and chemotherapy combined with radiation therapy [1.20 (1.17–1.23)], had a higher risk of mortality than those who did not undergo treatment. As the number of chronic diseases increased, the risk of mortality increased. Finally, patients who visited the emergency room had a higher risk of mortality than those who did not [1.35 (1.33–1.38)].

**Table 2:** Multivariate cox proportional hazards analysis of the development cohort.

Covariates		*N*	Mortality	IR^a^	HR (95% CI)					
					Model 1^b^	*p*- value	Model 2^c^	*p*- value	B regression coefficient	Point
Sex	Men	65,816	43,374 (65.9%)	25.6	1 (Ref.)		1 (Ref.)		1(Ref.)	
	Women	30,439	14,867 (48.8%)	14.5	0.60 (0.59–0.61)	<0.001	0.72 (0.71–0.74)	<0.001	−0.0965	−5
Age	19–44	3,538	1,408 (39.8%)	10.6	1 (Ref.)		1 (Ref.)		1(Ref.)	
	45–64	37,428	17,887 (47.8%)	14.0	1.29 (1.22–1.36)	<0.001	1.30 (1.23–1.38)	<0.001	0.0753	4
	≥65	55,289	38,946 (70.4%)	29.6	2.55 (2.41–2.69)	<0.001	2.32 (2.19–2.45)	<0.001	0.2667	15
Smoking status	Non	69,916	39,110 (55.9%)	18.4	1 (Ref.)		1 (Ref.)		1(Ref.)	
	Current	26,339	19,131 (72.6%)	32.1	1.63 (1.60–1.66)	<0.001	1.35 (1.33–1.38)	<0.001	0.088	5
Alcohol consumption	No	77,386	45,401 (58.7%)	20.1	1 (Ref.)		1 (Ref.)		1(Ref.)	
	Yes	18,869	12,840 (68.1%)	27.5	1.31 (1.28–1.33)	<0.001	1.08 (1.06–1.10)	<0.001	0.0226	1
Exercise	Non	60,011	38,093 (63.5%)	23.5	1 (Ref.)		1 (Ref.)		1(Ref.)	
	Regular	36,244	20,148 (55.6%)	18.3	0.80 (0.79–0.82)	<0.001	0.85 (0.83–0.86)	<0.001	−0.0539	−3
Income	Others	79,417	47,632 (60.0%)	21.0	1 (Ref.)		1 (Ref.)		1(Ref.)	
	Low	16,838	10,609 (63.0%)	23.1	1.09 (1.07–1.11)	<0.001	1.07 (1.05–1.09)	<0.001	0.0221	1
BMI (kg/m^2^)	<18.5	4,672	3,369 (72.1%)	31.4	1.37 (1.32–1.42)	<0.001	1.16 (1.12–1.21)	<0.001	0.0588	3
	18.5–25	62,828	38,580 (61.4%)	22.0	1 (Ref.)		1 (Ref.)		1(Ref.)	
	≥25	28,755	16,292 (56.7%)	18.9	0.87 (0.85–0.89)	<0.001	0.90 (0.89–0.92)	<0.001	−0.0349	−2
Treatment	None	33,927	21,485 (63.3%)	26.4	1 (Ref.)		1 (Ref.)		1(Ref.)	
	Chemotherapy	7,652	6,262 (81.8%)	36.8	1.24 (1.21–1.28)	<0.001	1.24 (1.21–1.28)	<0.001	0.2067	11
	Surgery	15,381	2,525 (16.4%)	3.7	0.15 (0.14–0.16)	<0.001	0.16 (0.16–0.17)	<0.001	−0.3975	−22
	Radiation	16,092	13,086 (81.3%)	42.3	1.43 (1.40–1.46)	<0.001	1.25 (1.22–1.28)	<0.001	0.1585	9
	Surgery + chemotherapy	2,136	770 (36.1%)	8.5	0.34 (0.31–0.36)	<0.001	0.36 (0.34–0.39)	<0.001	−0.2082	−12
	Surgery + radiation	4,863	2,199 (45.2%)	12.3	0.48 (0.46–0.50)	<0.001	0.45 (0.43–0.47)	<0.001	−0.1589	−9
	Chemotherapy + radiation	12,358	10,177 (82.4%)	32.3	1.09 (1.07–1.12)	<0.001	1.20 (1.17–1.23)	<0.001	0.2484	14
	Surgery + chemotherapy + radiation	3,846	1,737 (45.2%)	11.1	0.43 (0.41–0.46)	<0.001	0.49 (0.46–0.51)	<0.001	−0.1037	−6
Number of chronic diseases	0	2,273	1,014 (44.6%)	12.6	1 (Ref.)		1 (Ref.)		1 (Ref.)	
	1	5,940	3,011 (50.7%)	15.3	1.20 (1.11–1.28)	<0.001	1.07 (1.00–1.15)	0.1	0.0181	1
	2	9,203	5,013 (54.5%)	17.5	1.34 (1.26–1.44)	<0.001	1.13 (1.05–1.21)	0	0.0297	2
	3	11,614	6,680 (57.5%)	19.3	1.48 (1.38–1.58)	<0.001	1.18 (1.11–1.26)	<0.001	0.0408	2
	4	12,820	7,509 (58.6%)	20.1	1.53 (1.43–1.64)	<0.001	1.20 (1.12–1.28)	<0.001	0.0444	2
	≥5	54,405	35,014 (64.4%)	24.3	1.81 (1.70–1.93)	<0.001	1.30 (1.22–1.39)	<0.001	0.0716	4
Emergency room visit	No	65,683	35,307 (53.8%)	17.9	1 (Ref.)		1 (Ref.)		1(Ref.)	
	Yes	30,572	22,934 (75.0%)	30.7	1.56 (1.54–1.59)	<0.001	1.28 (1.26–1.31)	<0.001	0.1303	7

HR, hazard ratio; IR, incidence rate. ^a^Incidence per 1,000 person-years. ^b^Model 1 was not adjusted. ^c^Model 1 was not adjusted.

In the risk scoring system, participants aged ≥65 years had the highest risk score (15 points), followed by those that underwent combined chemotherapy and radiation therapy (14 points), chemotherapy (11 points), and radiation therapy (9 points), those with a history of emergency room visits (7 points), and current smokers (5 points). Participants aged 45–64 years and those with ≥5 chronic diseases each scored 4 points. Additionally, participants treated by surgery had the lowest risk score (−22 points), followed by those treated by surgery combined with chemotherapy (−12 points), participants treated by surgery combined with radiation therapy (−9 points), those that underwent surgery combined with chemotherapy and radiation therapy (−6 points), women (−5 points), regular exercisers (−3 points), and those with a BMI ≥25 kg/m^2^ (−2 points).

### Validation of the risk scoring system in lung cancer

**Figure 1:**
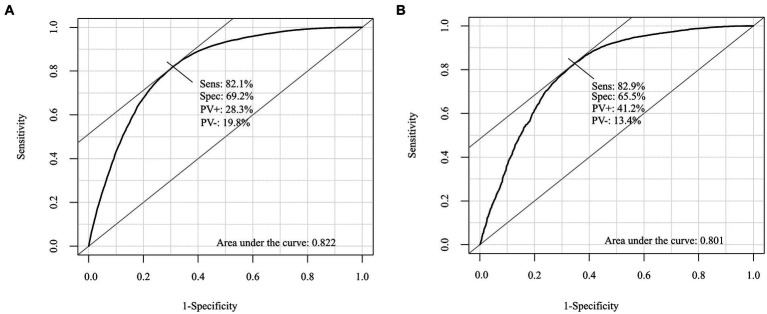
Receiver operating characteristic (ROC) curve for the development and validation cohorts. **(A)** The development cohorts. **(B)** The validation cohorts.

**Figure 2:**
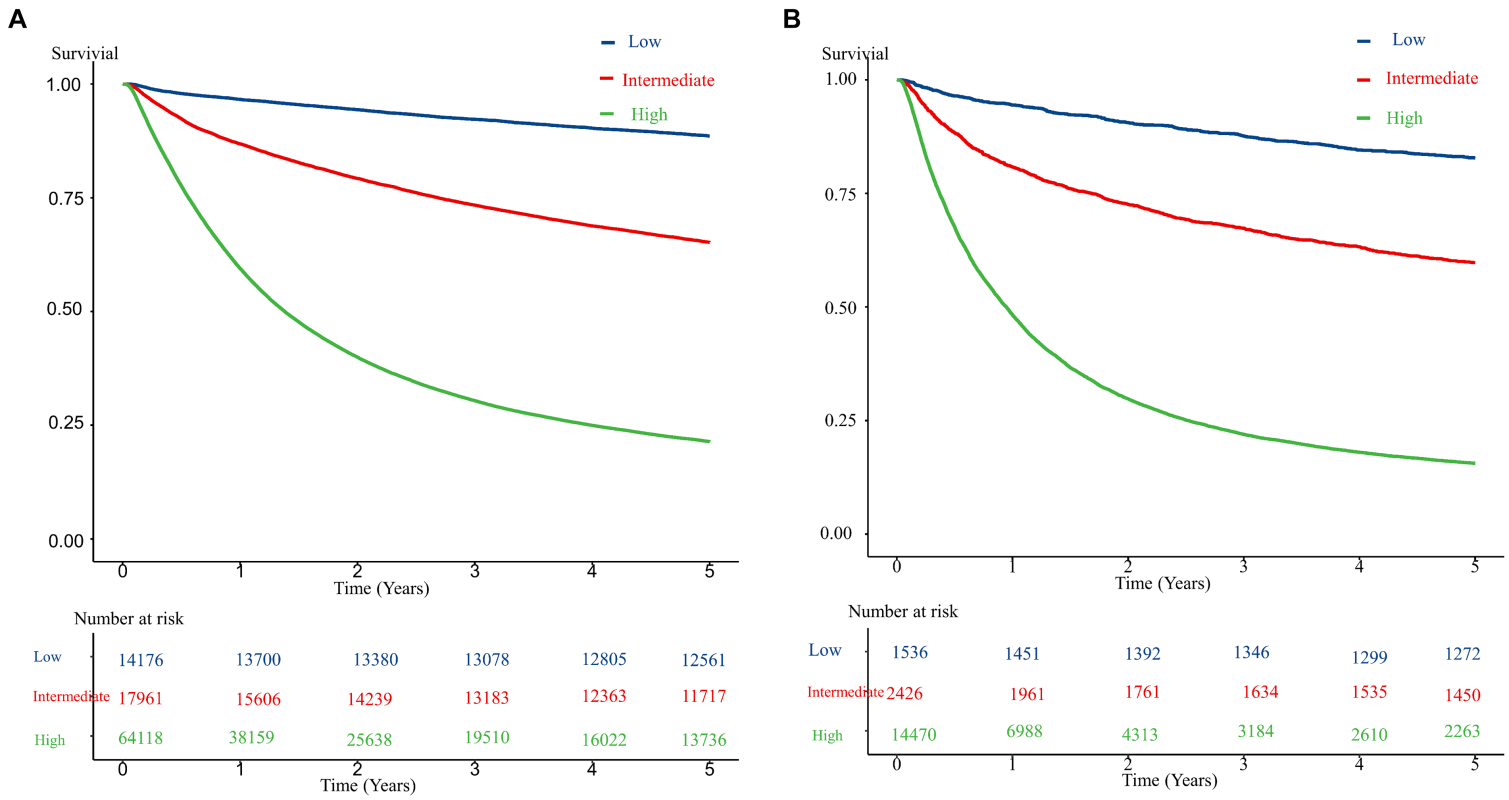
Kaplan–Meier survival curve for the development and validation cohorts according to risk level. **(A)** The development cohorts. **(B)** The validation cohorts.

Figure 1 presents the ROC curves for the development and validation cohorts. The area under the curve was 0.82 in the development cohort and 0.80 in the validation cohort. These values show the high discriminative ability of our risk scoring model. Table 3 presents the risk of mortality at 1, 3, and 5 years in the development and validation cohorts according to the mortality risk category. Among both cohorts, as the risk category increased, the percentage of the risk of mortality increased in all time periods. The C statistics in the development and validation cohorts were 0.78 (0.77–0.78) and 0.81 (0.78–0.84), respectively. As shown in the Kaplan–Meier survival curves according to risk category, the survival rate decreased as the follow-up period increased, and the slope of the graph became steeper from the low-risk to high-risk group (Figure 2).

**Table 3:** Risk of mortality at one, three, and 5 years in the development and validation cohorts.

Risk category	Development cohort (Seoul)				Validation cohort (Busan and Gyeongsangnam-do)			
		Percentage of mortalities (95% CI)						
		At 5 years	At 1 year	At 3 years		At 5 years	At 1 year	At 3 years
Low	14,176 (14.7%)	11 (10–12)	3 (2–4)	8 (7–9)	1,536 (8.3%)	17 (10–24)	5 (4–7)	12 (8–16)
Intermediate	17,961 (18.7%)	35 (33–36)	13 (11–15)	26 (25–28)	2,426 (13.2%)	40 (36–44)	19 (15–23)	33 (29–37)
High	64,118 (66.6%)	78 (77–79)	40 (39–41)	69 (68–70)	14,470 (78.5%)	84 (83–86)	52 (50–54)	78 (75–81)
		Difference in probability of mortality^a^			Difference in probability of mortality^a^			
		0.67	0.37	0.61		0.67	0.47	0.66
C statistic^b^ (95% CI)		0.78 (0.77–0.78)				0.81 (0.78–0.84)		

^a^The difference in the probability of mortality between the high- and low-risk groups was calculated as (Phigh-Plow)/100. ^b^The C statistic for the overall score is presented.

## Discussion

Our study showed the risk and preventive factors for mortality and developed a risk scoring system for mortality among patients with lung cancer. We also performed external validation of our system using nationwide cohort data in Korea. Advanced age, some types of treatment, and emergency room visits were the strongest risk factors of mortality, while other types of treatment, being female, regular exercise, and obesity were preventive factors of mortality. According to the external validation methods, our risk scoring system accurately predicted the mortality risk of Korean patients with lung cancer.

Our study showed that 10 variables were associated with the risk of mortality in patients with lung cancer. In Thailand, among 17,687 patients with lung cancer that had been admitted to the intensive care unit, the risk of 1-year mortality was increased by 3 and 22%, respectively, in those aged 65–74 years and ≥ 75 years compared to those aged 18–64 years.^5^ Other studies have estimated that the elderly tend to treat and investigate potential illnesses less than younger people because physicians and patients are often less adherent to guidelines in this population (12). Consistent with other risk assessment studies (13, 14), an emergency room visit increased the risk of mortality because it occurred due to general weakness, exacerbation of chronic diseases and lung cancer, and infection. Moreover, current smokers had an independently increased risk of mortality, perhaps because smoking decreases the lung function and lung volume and can worsen chronic diseases (15). In addition, smoking is the strongest risk factor of lung cancer and is associated with cardiovascular and other pulmonary diseases (16). In another study, the hazard ratio of moderate and severe comorbidity ranged from 1.04 to 1.78 compared to no and mild comorbidity among lung cancer patients (17). Cancer patients with severe comorbidity were associated with an increased risk morbidity (18). Finally, individuals with many chronic diseases experience a decreased quality of life, greater use of medical facilities, and decreased physical activity (19, 20).

According to previous studies, the mortality risk of lung cancer differs depending on the type of anticancer treatment (6, 21, 22). Because the lung cancer stage and biopsy were not included in the KNHIS database, we analyzed the type of anticancer treatment. The mortality risk was lowest when patients were treated by surgery, which is consistent with another study (6). Similarly, surgery has been found to be among the best treatment strategies for non-small cell lung cancer stage IA to IIB and limited-stage small cell lung cancer (23). Therefore, patients treated by surgery had a lower mortality risk because they were in the early stages of disease. On the other hand, advanced lung cancer patients tended to undergo chemotherapy (23). A meta-analysis showed that high BMI decreased mortality risk in patients with lung cancer. Specifically, a BMI increase of 5 kg/m^2^ decreased the mortality risk by 12% (24). Consistent with those results, our study found that underweight patients experienced an increased mortality risk, while those with obesity had a decreased mortality risk.

Being a woman and regular exercise were the main preventive factors of lung cancer-related mortality. Unlike the pattern in other countries, in Korea, the prevalence of lung cancer among men was much higher than that among women (3) because of the significant difference between smoking habits in men and women (25). Previous studies have shown that screening tests (26) and healthy smoking- and alcohol-related behaviors (27) affect cancer-related mortality among men and women. Furthermore, among 38,000 American men, high- and moderate-intensity exercise resulted in a 57 and 52% lower mortality risk than low-intensity exercise, because exercise may improve immune function and systemic inflammation, decrease oxidative stress, and improve pulmonary function (28).

Risk stratification using our risk scoring model could identify at-risk patients and decrease the risk of mortality. Because our model provides comprehensive risk assessment including BMI, income, health behavior, and healthcare use, it can be used for managing the treatment of patients with lung cancer. We used multivariate analysis to confirm the risk factors of lung cancer. In addition, our risk scoring model was validated using an independent external cohort.

Despite the advantages of our study, it had some limitation. First, the KNHIS database is used for prescription purposes, and chronic diseases might be over-diagnosed or under-diagnosed if the diagnosis codes were unclear. In addition, the KNHIS did not include the stage and biopsy results of lung cancer, which is the most important prognostic factor of cancer; therefore, it was adjusted by the type of treatment. In addition, because we did not used cancer registration data, the exact incidence rate and primary cancer status cannot be unclear. Third, although we considered many confounders that could affect mortality among patients with lung cancer, we did not include confounders that were not included in the KNHIS, such as the care provider and pulmonary function. Finally, because the present study was conducted on the population of only one country, we were unable to establish a completely different validation cohort from the development cohort. Despite the limitations, we identified risk and preventive factors of mortality among patients with lung cancer and validated our risk scoring system using an external validation cohort. Therefore, this study could be helpful in identifying patients’ likelihood of survival.

In conclusion, we developed a risk scoring system to predict the risk of mortality among patients with lung cancer. Advanced age, cancer stage, and some types of anticancer treatment were strong risk factors of mortality in patients with lung cancer. In contrast, being female, some types of anticancer treatment, and exercise were preventive factors of mortality in patients with lung cancer. These results will aid clinicians in predicting the risk of mortality and appropriately managing lung cancer patients.

## Data availability statement

The data analyzed in this study is subject to the following licenses/ restrictions: the dataset analyzed in the present study is available from the corresponding author upon reasonable request. Requests to access these datasets should be directed to KS, mdsky75@gmail.com.

## Ethics statement

This study adhered to the principles of the Declaration of Helsinki and was approved by the Institutional Review Board of Uijeongbu Eulji Medical Center (IRB no. UEMC 2021–08-022). The requirement for informed consent was waived by the Institutional Review Board of Uijeongbu Eulji Medical Center due to the use of anonymized and de-identified data.

## Author contributions

YH: Conceptualization, Data curation, Investigation, Methodology, Writing – original draft. H-RK: Data curation, Formal analysis, Methodology, Writing – review & editing. HK: Conceptualization, Investigation, Writing – review & editing. KS: Conceptualization, Data curation, Methodology, Supervision, Validation, Visualization, Writing – original draft, Writing – review & editing.
